# Outcome of patients with double valve surgery between 2009 and 2018 at University Hospital Basel, Switzerland

**DOI:** 10.1186/s13019-022-01904-9

**Published:** 2022-06-13

**Authors:** Martin L. Egger, Brigitta Gahl, Luca Koechlin, Lena Schömig, Peter Matt, Oliver Reuthebuch, Friedrich S. Eckstein, Martin T. R. Grapow

**Affiliations:** 1 Department of Cardiac Surgery, University Hospital Basel, University Hospital Basel, 4031 Basel, Switzerland; 2 University of Basel, 4051 Basel, Switzerland; 3 Herzchirurgie, Kantonsspital Luzern, 6000 Luzern, Switzerland; 4 HerzZentrum Hirslanden Zürich, 8008 Zurich, Switzerland

**Keywords:** Double valve replacement, Mitral valve repair, Mitral valve replacement

## Abstract

**Background:**

In isolated mitral valve regurgitation general consensus on surgery is to favor repair over replacement excluding rheumatic etiology or endocarditis. If concomitant aortic valve replacement is performed however, clinical evidence is more ambiguous and no explicit guidelines exist on the choice of mitral valve treatment. Both, double valve replacement (DVR) and aortic valve replacement in combination with concomitant mitral valve repair (AVR + MVP) have been proven to be feasible procedures. In our single-center, retrospective, observational cohort study, we compared the outcome of these two surgical techniques focusing on mortality and morbidity.

**Methods:**

89 patients underwent DVR (n = 41) or AVR + MVP (n = 48) in our institution between 2009 and 2018. Follow-up data was collected using electronic patient records, by contacting treating physicians and by telephone interviews. We used the Kaplan–Meier method to analyze mortality during follow-up and Cox regression to investigate potential predictors of mortality.

**Results:**

During a median follow-up duration of 4.5 [IQR 2.9 to 6.1] years, there was no significant difference in mortality between both cohorts. Thirty days mortality was 6.3% in the DVR and 7% in the AVR + MVP cohort. Overall mortality amounted to 17% for DVR and 23% for AVR + MVP. DVR was the preferred procedure for valve disease of rheumatic etiology and for endocarditis, while in degenerative valves AVR + MVP was predominant. More biological valves were used in the AVR + MVP cohort (*p* < 0.001) and more mechanical valves were implanted in the DVR cohort. The rate of rehospitalization, deterioration of left ventricular ejection fraction and postoperative complications were equally distributed among the two cohorts.

**Conclusion:**

Our data analysis showed that both DVR and AVR + MVP are safe and feasible options for double valve surgery. Based on our findings we could not prove superiority of one surgical technique over the other. Choosing the appropriate procedure for the patient should be influenced by valve etiology, patients’ comorbidities and the surgeons’ experience.

***Trial registration*:**

This was a retrospectively registered trial, registered on April 1st 2018, ClinicalTrials.gov Identifier: NCT03667274.

## Background

Mitral valve repair in patients with primary mitral regurgitation is the gold standard and in general a class I recommendation in US and European guidelines [1]. Over the last decades, evidence has accumulated that surgical treatment in isolated primary, nonrheumatic mitral valve regurgitation is indicated, mitral valve repair (MVP) should be favored over mitral valve replacement (MVR) due to lower overall mortality and postoperative morbidity [2]. However, concerning the surgical treatment of concomitant aortic and mitral valve disease, different approaches are available. Double valve replacement (DVR) is a well-established method with reasonable survival rates, although requiring strict anticoagulation regimens in many patients [3, 4]. Aortic valve replacement combined with mitral valve repair (AVR + MVP), a more recently developed entity, has also proven to be a feasible option [5], Some data suggests DVR to be the superior method. Hamamoto et al. found similar survival rates (DVR 81% vs. 79% AVR + MVP), but significantly lower reoperation rates on the mitral valve in a 15 year follow-up (DVR 46% vs. 85% AVR + MVP) [6]. Other studies have shown AVR + MVP to be associated with better survival. Gilinov et al. found a significantly better late survival with 46% for AVR + MVP versus 34% for DVR in a 15 year follow up in 813 patients [7]. Leavitt et al. confirmed this finding with a 9.1 year median survival for AVR + MVP versus 6.3 years in DVR [8]. European guidelines are yet to be established on the surgical treatment for multiple and mixed valve diseases, as currently available data appears to be insufficient for evidence based recommendations [9]. In concomitant aortic valve replacement it remains therefore contentious whether the mitral valve should be replaced or repaired.

In this retrospective, observational study, we included 89 patients who underwent double valve surgery with combined aortic and mitral valve disease for a period of 9 years between 2009 and 2018 at the Department of Cardiac Surgery at University Hospital Basel, Switzerland, comparing mortality and postoperative complications for each sub-group of DVR versus AVR + MVP. As approximately ten percent of patients with valvular heart disease suffer from combined aortic and mitral valve disease [7, 10], our aim was to achieve more evidence on this topic.

## Methods

### Patient population

**Fig. 1:**
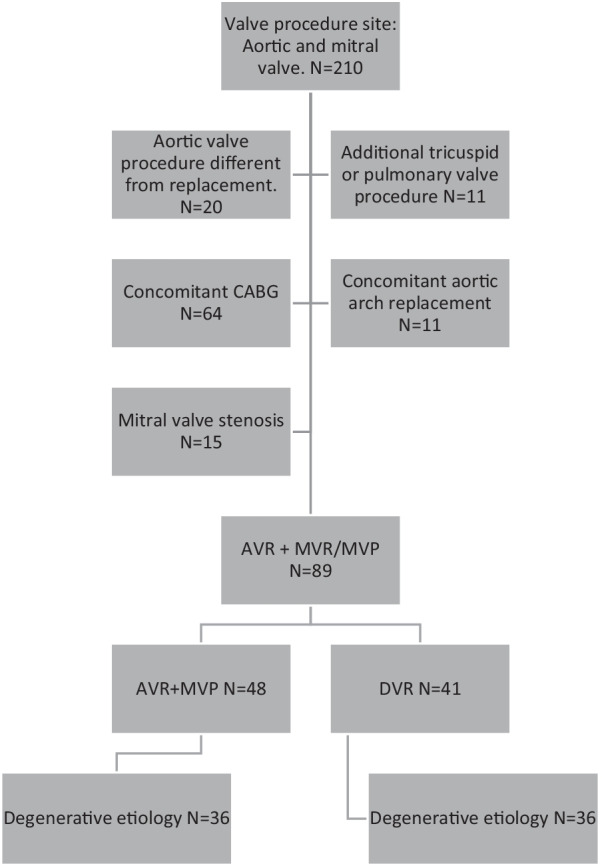
Flowchart of included patients

Using a prospectively maintained institutional registry (Intellect 1.7, Dendrite Clinical Systems, Henley-on-Thames, UK), we identified all consecutive patients who underwent double valve surgery on the aortic and mitral valve between 2009 and 2018 at the Department of Cardiac Surgery at University Hospital Basel, Switzerland. We excluded all patients who underwent any procedure different to replacement on the aortic valve, such as repair. In addition, all patients with concomitant surgery on tricuspid or pulmonary valve, with concomitant coronary artery bypass grafting (CABG) or concomitant aortic arch replacement were excluded to reduce confounders such as longer duration of operation or greater intraoperative hypothermia. A flow chart of our screened patients is provided in Fig. 1. Concerning the etiology of valve disease, we were particularly interested in the mitral valve. The specific cause of the valve defect, i.e. its’ etiology, was determined by preoperative diagnostics, such as clinical, echocardiographic or radiological information, intraoperative findings and postoperative pathology reports. We then excluded all patients presenting with mitral valve stenosis or with combined mitral valve disease to exclude valves where reconstruction is not a feasible surgical option. Further, we created a subgroup with all patients suffering from degenerative valve disease. Preoperative comorbidities of patients were not specified but were expressed in the risk model EuroSCORE II [11] which is routinely recorded to calculate the perioperative risk of death. The type of valve used to replace either the mitral or the aortic valve was defined as either mechanical or biological and was not further differentiated. For mitral valve repair we included annuloplasty, neochordae implantation, patch plasty and resection of endocarditic deposits.

### Outcomes

Our primary outcome was death during follow-up time. We classified cause of death as (i) cardiovascular, (ii) procedure related (i.e. death as a cause of a perioperative complication), (iii) other (i.e. sepsis, cerebral or pulmonary cause) or (iv) death of unknown cause. We defined early mortality as in-hospital death or death within the first 30 days postoperatively and late mortality as death beyond the first 30 days after surgery according to the “Guidelines for Reporting Mortality and Morbidity After Cardiac Valve Interventions” [12].

Secondary outcome included (i) postoperative complications (i.e. pericardial tamponade, thoracic hematoma, sternal infection, atrioventricular block and cardiogenic shock), (ii) reoperations on the aortic or mitral valve and (iii) rehospitalizations due to cardiac decompensation and left ventricular ejection fraction (LVEF) assessed by echocardiography.

### Data collection

Before data collection was initiated, ethical approval had to be applied for from the Ethikkommission Nordwest- und Zentralschweiz (EKNZ). On April 16th 2018, we finally received proxy consent for further use of patient data for this study.

Baseline and perioperative data was acquired through the prospectively maintained institutional registry (Intellect). Survival data and secondary outcomes were retrospectively obtained through outpatient visits or readmissions to University Hospital Basel by exploration of the dedicated in-house database. In addition, we mailed specific questionnaires to cardiologists, last known treating physicians or clinics of last hospitalization. If neither electronic database records nor the questionnaires would provide sufficient data information, patients or their close relatives were interviewed by telephone.

### Statistical analysis

The primary outcome—death during follow-up time—was analyzed in a time-to-event manner. We used the Kaplan–Meier method for visualization and performed a log rank test. To investigate the impact of age, comorbidities, surgical procedure and preoperative leading disease during follow-up on mortality, we used Cox regression and checked proportional hazard assumption using Schoenfeld residuals. Continuous variables were shown as median and interquartile range, categorical variables as numbers with percentage. All analyses were carried out using Stata 15 (Stata Corp., College Station, Texas).

## Results

### Baseline characteristics

210 were initially screened. We finally included a total cohort of 89 patients in our study.

**Fig. 2:**
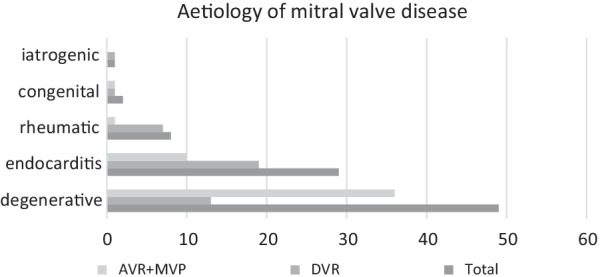
Etiology of mitral valve disease in our patient cohort

A detailed overview of patient characteristics is presented in Table 1. The median age of the AVR + MVP was 68 years [IQR 62 to 75] vs. 65 years in the DVR group [IQR 57 to 72] (*p* = 0.18). The majority of our patients was male with 65% of our total population. Estimated median risk of according to EuroSCORE II was 5.9% [IQR 2.9 to 12] for the AVR + MVP group and 4.6% [IQR 2.8 to 12] for the DVR group (*p* = 0.67). As shown in Fig. 2, mitral valve etiology was frequently degenerative (N = 49) and 32.5% of patients presented with endocarditis (N = 29). While more patients (*p* = 0.002) with degenerative valve disease underwent AVR + MVP than did DVR, more patients with endocarditis underwent DVR.

**Table 1:** Baseline characteristics

	DVR (N = 41)	AVR + MVP (N = 48)	*p*
Age	65 [57 to 72]	68 [62 to 75]	0.18
Age ≥ 70 years	13 (32%)	18 (38%)	0.66
*Gender*			0.34
Female	14 (34%)	11 (23%)	
Male	27 (66%)	37 (77%)	
EuroSCORE II	4.6 [2.8 to 12]	5.9 [2.9 to 12]	0.67
*Comorbidities*			
Vascular disease	9 (22%)	5 (10%)	0.13
Diabetes	5 (12%)	6 (13%)	0.96
COPD	4 (10%)	1 (2%)	0.11
Kidney disease	13 (31%)	18 (38%)	0.56
Atrial fibrillation	20 (49%)	29 (60%)	0.27
Hypertension	8 (20%)	14 (29%)	0.29
Obesity	7 (17%)	5 (10%)	0.36
Hepatic cirrhosis	2 (5%)	0%	0.20
*AV_etiology*			0.002
Degenerative	15 (37%)	34 (71%)	
Congenital	1 (2.4%)	1 (2.1%)	
Paravalvular leak	0 (0.00%)	1 (2.1%)	
Rheumatic	7 (17%)	1 (2.1%)	
Endocarditis	18 (44%)	11 (23%)	
Active endocarditis	13 (72%)	7 (64%)	0.63
Chronic endocarditis	5 (28%)	4 (36%)	0.63
*AV_disease*			0.10
Stenosis	6 (15%)	13 (27%)	
Regurgitation	31 (76%)	27 (56%)	
Combined	4 (10%)	4 (8.3%)	
Abscess	0 (0.00%)	4 (8.3%)	
*MV_etiology*			< 0.001
Degenerative	13 (32%)	36 (75%)	
Congenital	1 (2.4%)	1 (2.1%)	
Rheumatic	7 (17%)	1 (2.1%)	
Iatrogenic	1 (2.4%)	0 (0.00%)	
Endocarditis	19 (46%)	10 (21%)	
*MV_disease*			0.25
Regurgitation	41 (100%)	44 (92%)	
Abscess	0 (0.00%)	3 (6.3%)	
Perforation	0 (0.00%)	1 (2.1%)

All 89 patients were operated by five senior surgeons. The surgical approach was in all cases partial or conventional sternotomy. A systematic listing of surgery details can be viewed in Table 2. The type of valve used for replacement was defined as biological or mechanical. More biological valves (87%) were used for the aortic valve in the AVR + MVP group (*p* < 0.001) compared to the DVR group (51%). The duration of the intervention was significantly longer in the DVR group (mean = 240 min) compared to the AVR + MVP group (mean = 220 min).

**Table 2:** Surgery details

	DVR (N = 41)	AVR + MVP (N = 48)	*p*
Emergency	1 (2.4%)	2 (4.2%)	1.00
Duration of operation	240 [225 to 270]	220 [180 to 244]	0.010
Perfusion time in min	165 [142 to 212]	157 [126 to 185]	0.19
Clamping time in min	127 [109 to 159]	117 [93 to 146]	0.12
*Procedure groups*			0.64
Valve(s) and other	11 (27%)	16 (33%)	
Valve(s) only	30 (73%)	32 (67%)	
*Type of AVR*			< 0.001
Biological	21 (51%)	42 (87%)	
Mechanical	20 (49%)	6 (13%)	
*Type of MVR*			
Biological	22 (54%)		
Mechanical	19 (46%)		

### Follow-up

Median follow-up was 4.5 years [IQR 2.9 to 6.1 years], longest observation time was 10.8 years. As shown in Table 3, median follow-up in the DVR group was 4.6 years and 4.5 years in the AVR + MVP cohort.

**Table 3:** Assessments and outcomes during follow-up

	DVR (N = 41)	AVR + MVP (N = 48)	*p*
Years of follow-up	4.6 [3.0 to 6.1]	4.5 [2.8 to 6.0]	0.65
Postoperative LVEF (%)	51 [40 to 60]	48 [40 to 55]	0.11
Three months LVEF %	46 [35 to 54]	48 [35 to 60]	0.56
Twelve months LVEF	42 [29 to 55]	54 [41 to 58]	0.45
Postoperative complications	7 (17%)	5 (10%	0.36
Rehospitalisation due to cardiac cause	3 (7%)	7 (14.5%)	0.28
Reoperation on aortic and/or mitral valves	2 (4.9%)	1 (2.0%)	0.47
Thirty days mortality	3 (7%)	3 (6.3%)	0.84
Thirty days mortality (excluding endocarditis)	2 (4.8%)	1 (2%)	0.46
Overall Death	7 (17%)	11 (23%)	0.60
*Cause of death*			1.00
Cardiovascular	1 (2.4%)	3 (6.3%)	
Other	4 (10%)	5 (10%)	
Unknown	2 (4.6%)	3 (6.3%)	

**Fig. 3:**
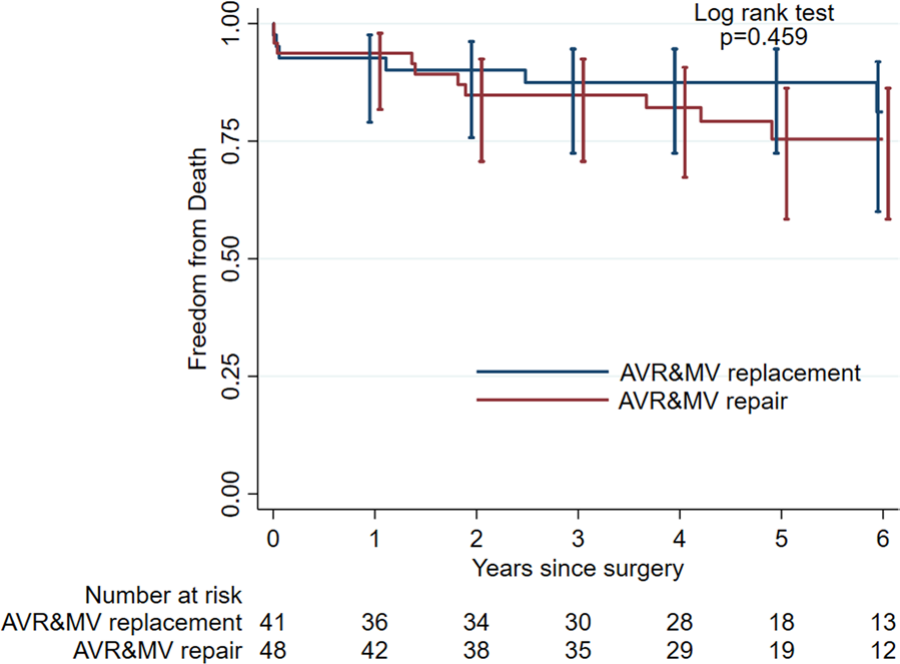
Kaplan–Meier survival estimates for DVR and AVR + MVP

**Fig. 4:**
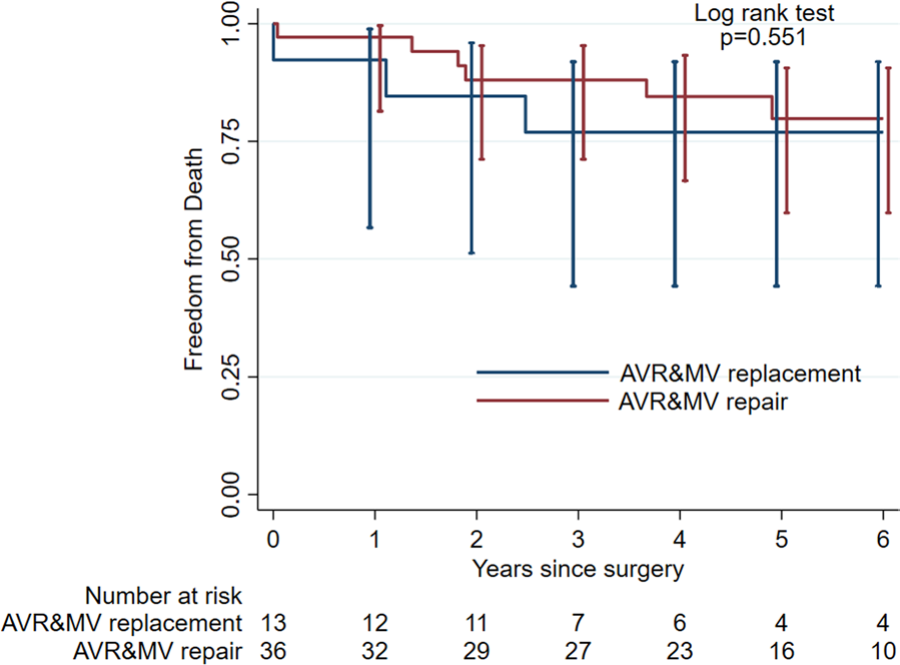
Kaplan–Meier survival estimates for DVR versus AVR + MVP in patients with degenerative mitral etiology

Our primary outcome was death during follow-up. Mortality rate over 405 patient years was 4.4 per 100 patients per year (95% CI 2.8 to 7.0) during the whole follow-up period. Thirty days mortality was equal in both groups at n = 3, which represented 7% in the DVR and 6.3% in the AVR + MVP cohort (*p* = 0.84). Thirty days mortality when excluding endocarditis was reduced to n = 1 (2%) for AVR + MVP and n = 2 (4.8%) for DVR (*p* = 0.46). Figure 3 depicts survival estimates derived by applying the Kaplan–Meier method. Figure 4 shows survival estimates of patients with degenerative etiology of the valve disease. During follow-up, we calculated the hazard of death within 6 years associated with MVP. In our cohort MVP did not have a clear impact on survival as compared to MVR overall and in degenerative etiology, as Hazard Ratio (HR) of MVP for death was slightly below 1 without reaching statistical significance. This result was robust with respect to adjustment for comorbidities (EuroSCORE II), patient age ≥ 70 years, use of mechanical aortic valve or endocarditis, as well as combinations. When adjusted for perfusion time, HR of MVP increased slightly above one. As expected EuroSCORE II and perfusion time were associated with a higher HR. Perfusion time, however, violated proportional hazard assumptions when included into the model. In summary, our data did not show any statistically significant survival benefit during follow-up in either of the two surgical procedures compared to the other. Our data did not exhibit any survival difference of the two surgical strategies.

Concerning our secondary outcomes, left ventricular ejection fraction (LVEF) assessed by preoperative echocardiography during the postoperative hospitalization and at 3 and 12 months postoperatively also showed no statistically significant difference between the two groups. 17% of patients in the DVR cohort and 10% in the AVR + MVP group developed postoperative complications. 3 Patients (7.3%) after DVR and 2 (4.0%) patients after AVR + MVP developed atrioventricular block. One patient each (2.4%) in the DVR group presented with retrosternal hematoma, pericardial tamponade and septic or cardiogenic shock. In the AVR + MVP cohort, 2% of patients presented with either pleural effusion, retrosternal hematoma or retrosternal abscess. In total, three patients had to be reoperated on the aortic and/or the mitral valve. Two patients in the DVR cohort (4.9%) were reoperated due to either paravalvular leak or acute endocarditis and one patient (2%) in the AVR + MVP group was reoperated because of degeneration of a biological valve. Rehospitalization rates in the follow-up period due to a cardiac cause, such as cardiac decompensation or symptomatic arrhythmia, were higher in the AVR + MVP cohort with 14.5% compared to 7% in the DVR cohort, although statistical significance was not reached (*p* = 0.28).

## Discussion

Our single-center, retrospective observational cohort study of 89 patients that had undergone a concomitant surgical aortic and mitral valve procedure showed an overall survival rate of 78%, which comparatively is an excellent survival rate during the total follow-up period of 2.5 to 6.1 years. Our data therefore suggests that aortomitral valve surgery is a feasible and safe option, irrespective of which method is used, considering the comorbidities of these patients. Our data showed a low valve reoperation rate (3.3%), low complication rate (13.5%), rehospitalization rate (11.2%) and low morbidity overall.

The primary outcome of our study was mortality during follow-up. Our data showed equal survival after DVR compared to AVR + MVP, when either surgery was performed from 2009 to 2018 at the Department of Heart Surgery at University Hospital Basel. Thirty days postoperative mortality was 6.7% and equally distributed among the two cohorts. Difference in mortality overall showed no statistical significance, while being slightly higher in the AVR + MVP group at 23% compared to 17% in the DVR cohort. Whilst there was no statistical evidence of better survival for one procedure over the other in our study, the graphical presentation in our Kaplan–Meier estimate shown in Fig. 3 suggests a slight survival benefit for DVR. No previously published data could however support this graphical impression. It is doubtful if this effect would augment in case of a longer follow-up period in this patient cohort. Concerning our secondary endpoints reoperation rates, postoperative complications, rehospitalizations and postoperative LVEF remained equal among the two groups. We could however show that higher preoperative comorbidities and longer perfusion time were—as expected—associated with a higher HR for death.

It is noteworthy that these results remained equal even when restricting the analysis to patients with degenerative valve disease as we expected the inclusion of endocarditis and rheumatic valve etiology to have an impact on outcome. Considering our comparatively small study cohort it was not unexpected that the statistical analysis of the overall mortality did not detect any difference in the two cohorts either in 30 day or overall mortality.

Based on our study examining 89 patients with the primary and secondary outcomes being postoperative survival and morbidity, our data does not suggest a clinical difference in outcome and there is no clear evidence to be deduced therefrom whether one surgical method should be preferred. In summary, we therefore cannot generally recommend one procedure over the other.

The major strengths of our study were the next to equal size of treatment groups and their similar comorbidities compared to other studies. In addition, the etiology of the valve pathology was well documented and was thus taken into account for the evaluation of our results.

Several retrospective observational studies supporting our findings—i.e. that survival does not differ—have been published previously [13].

A multicenter retrospective study conducted in northern New England and published in 2009 observed 1057 patients found a significantly longer survival for AVR + MVP comparing DVR (9.1 vs. 6.3 years), but found a very high in-hospital mortality (15.5%) for double valve surgery in general. However, the study’s patient population was relatively old (45.9% > 70 years old), had a high rate of congestive heart disease (60.7%) and concomitant CABG was not excluded [8]. Our study found a significantly lower 30d mortality at 6.7% overall in a younger population (35% > 70 years old).

A study published in 2007 observed aortomitral valve surgeries with rheumatic etiology exclusively. Their statistics showed a slightly better survival for AVR + MVP with a 95% survival rate at 60 months postoperatively compared to 81% for DVR, which did not reach statistical significance. Their study cohort however was very unequal in distribution with 80% of patients receiving DVR which confirms our observation that DVR is widely considered to be the preferred treatment for rheumatic heart disease as 87.5% of our patients with rheumatic etiology received DVR. Albeit replacement may be more amenable for rheumatic valve disease due to fibrosis as scarring and sub-valvular pathology caused by this disease frequently prevent successful mitral valve repair, MVP was nevertheless chosen as a treatment option [5].

A much-cited retrospective study dated 2003 by Gillinov et al. showed similar postoperative mortality rates in both groups in a cohort consisting of 813 patients overall but found a significantly better late survival for AVR + MVP within 15 years with 46% survival for AVR + MVP versus 34% for DVR. The authors clearly promote mitral valve repair whenever possible, when concomitant AVR is performed due to better late survival. It is noteworthy that this study includes data from 1975 to 1998 and changes in surgical techniques, perioperative management and general advances in modern medicine (particularly cardiothoracic surgery) during the time of data collection have not been taken into account which may have affected the outcome as well [7].

Furthermore, a Japanese single-center study from 2003 with comparable patient populations showed no difference in survival (DVR 81% vs. 79% AVR + MVP) in the two cohorts but found a higher reoperation rate in their AVR + MVP group (DVR 46% vs. 85% AVR + MVP) with similar morbidity and therefore promotes DVR with mechanical valves whenever possible [6]. These findings deviate from ours as we noted nearly equal reoperation rates in our much shorter follow-up period of 4.5 years (DVR 4.9% vs. 2.0% AVR + MVP).

A more recent observational study published in 2014 observed 261 patients found no difference in survival in an 8 year follow up (79.8% for AVR + MVP vs. 75.2 for DVR). Patients older than 65 years benefited from AVR + MVP due to better survival (AVR + MVP: 9.4 years vs. DVR: 6.3 years). AVR + MVP showed better survival in non-rheumatic valve pathology, coinciding with our finding of 87.5% DVR in rheumatic etiology [14]. Coutinho et al. reported a similar double-center observational study in 2018 that observed 1122 patients operated in Portugal and Spain. This study showed higher 30 day mortality in DVR (4.2% vs. 1.8% in AVR + MVP) as well as better long-term survival for AVR + MVP at 12 years with a survival rate of 61.7% versus 53.3% in the DVR group. While our 30d mortality overall was higher (6.7%), distribution among the two cohorts was equal [15].

Finally a paper published in 2015 examining data from 41,417 patients from the U.S. Nationwide Inpatient Sample (NIS) found lower in-hospital mortality (10.1% for DVR vs. 7.9% in AVR + MVP), shorter hospitalization rates and lower costs with AVR + MVP compared to DVR [16].

As the above-mentioned studies on this topic show, the evidence is not conclusive yet, on whether DVR or AVR + MVP should be preferred in aortomitral valve surgery. Most study designs were observatory, retrospective or meta-analyses with, in most cases, heterogenous patient cohorts, both numerically and comparing patient characteristics and duration of follow-up period. It is therefore obvious that only vague recommendations exist and no clear evidence-based guidelines on mixed and multiple valvular heart surgery have been established by international societies [1, 14]. While randomized controlled trials would certainly help gain evidence in the matter, it remains debatable whether such a study would be possible from an ethical viewpoint. Based on our findings that there was no difference in mortality and morbidity however, it is suggestive that such a study could be conducted in a carefully selected study group.

### Limitations

Our main limitation consists of our comparatively small patient cohort. Secondly, bias of this study is probable as this is a retrospective observatory and a non-randomized study. Concomitant CABG was excluded due to its significant impact on morbidity and survival shown in previous studies and might limit comparison to other studies [8, 17]. We did not exclude rheumatic valve disease, which has been associated with a high risk of secondary valve replacement after primary repair and might have influenced our surgeons’ decision [7]. Lastly, our patient group was very heterogenous as we did not exclude active endocarditis.

## Conclusion

In this observational single-center retrospective study, we observed a mid-term overall survival rate of 78%. There was no difference in 30 day or long-term mortality between DVR versus AVR + MVP. Apart from a significantly higher reoperation rate with DVR, we could not find any superiority comparing survival, adverse event-free survival, rate of rehospitalization or functional deficiencies postoperatively in both cohorts. Unlike other studies conducted in this field, neither age, nor the use of either biological or mechanical valve showed any significant benefit in outcomes. As expected, longer perfusion time and more severe comorbidities lead to a higher HR. Our data suggests however that the preferred surgical treatment of combined aortic and mitral valve disease should be determined by underlying comorbidities, valve pathology and the surgeons clinical experience and should only secondarily be impacted by the patients’ age, i.e. on a case-by-case basis.

## Data Availability

The datasets used and/or analyzed during the current study are available from the corresponding author upon reasonable request.

## Abbreviations

DVR: Double valve replacement
AVR: Aortic valve replacement
MVP: Mitral valve repair
AVR + MVP: Aortic valve replacement with concomitant mitral valve repair
IQR: Interquartile range
MVR: Mitral valve replacement
CABG: Coronary artery bypass grafting
LVEF: Left ventricular ejection fraction
HR: Hazard ratio
EKNZ: Ethikkommission Nordwest- und Zentralschweiz
AV: Aortic valve
MV: Mitral valve
